# RSAP-Net: joint optic disc and cup segmentation with a residual spatial attention path module and MSRCR-PT pre-processing algorithm

**DOI:** 10.1186/s12859-022-05058-2

**Published:** 2022-12-06

**Authors:** Yun Jiang, Zeqi Ma, Chao Wu, Zequn Zhang, Wei Yan

**Affiliations:** Department of Computer Science and Engineering, Northwest Normal University, Lanzhou, China

**Keywords:** Joint optic disc and cup segmentation, Glaucoma screening, Convolutional neural work, Attention mechanism, Pre-processing, Retinex theory

## Abstract

**Background:**

Glaucoma can cause irreversible blindness to people’s eyesight. Since there are no symptoms in its early stage, it is particularly important to accurately segment the optic disc (OD) and optic cup (OC) from fundus medical images for the screening and prevention of glaucoma. In recent years, the mainstream method of OD and OC segmentation is convolution neural network (CNN). However, most existing CNN methods segment OD and OC separately and ignore the a priori information that OC is always contained inside the OD region, which makes the segmentation accuracy of most methods not high enough.

**Methods:**

This paper proposes a new encoder–decoder segmentation structure, called RSAP-Net, for joint segmentation of OD and OC. We first designed an efficient U-shaped segmentation network as the backbone. Considering the spatial overlap relationship between OD and OC, a new Residual spatial attention path is proposed to connect the encoder–decoder to retain more characteristic information. In order to further improve the segmentation performance, a pre-processing method called MSRCR-PT (Multi-Scale Retinex Colour Recovery and Polar Transformation) has been devised. It incorporates a multi-scale Retinex colour recovery algorithm and a polar coordinate transformation, which can help RSAP-Net to produce more refined boundaries of the optic disc and the optic cup.

**Results:**

The experimental results show that our method achieves excellent segmentation performance on the Drishti-GS1 standard dataset. In the OD and OC segmentation effects, the F1 scores are 0.9752 and 0.9012, respectively. The BLE are 6.33 pixels and 11.97 pixels, respectively.

**Conclusions:**

This paper presents a new framework for the joint segmentation of optic discs and optic cups, called RSAP-Net. The framework mainly consists of a U-shaped segmentation skeleton and a residual space attention path module. The design of a pre-processing method called MSRCR-PT for the OD/OC segmentation task can improve segmentation performance. The method was evaluated on the publicly available Drishti-GS1 standard dataset and proved to be effective.

## Background

Glaucoma is a chronic progressive optic neuropathy and one of the main causes of irreversible vision loss in the world [1]. According to the World Vision Report published by the World Health Organization in October 2019 [2], an estimated 64 million people worldwide have glaucoma, of which 6.9 million (10.9%) have moderate or severe distance vision impairment or blindness caused by more serious eye diseases.Glaucoma has no apparent symptoms in the early stages of its onset, and the resulting loss of vision is irreversible. Therefore, early screening and diagnosis of glaucoma are essential to prevent vision loss caused by glaucoma.

**Fig. 1:**
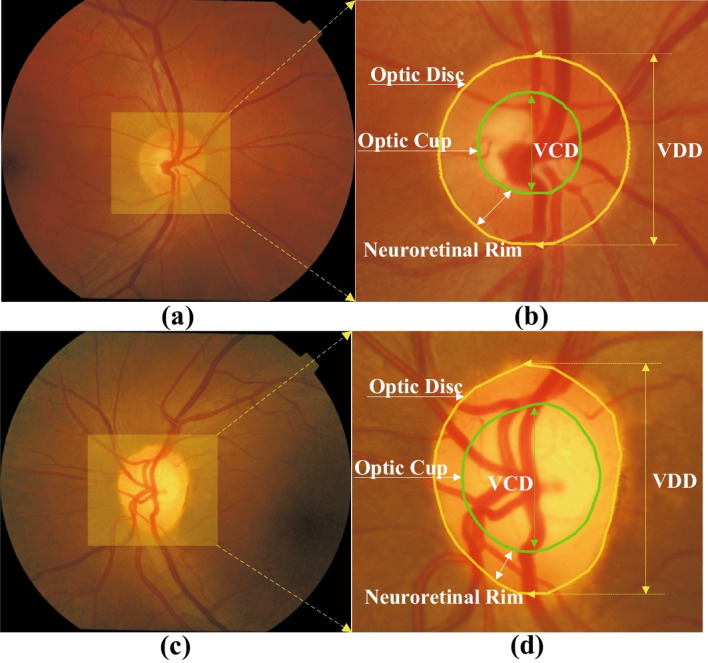
**a** Normal eye fundus image, **b** Normal eye’s enlarged OD and OC structure, **c** Glaucoma eye fundus image, **d** Glaucoma’s enlarged OD and OC structure

Currently, three common diagnostic modalities for glaucoma are optic nerve head (ONH) assessment [3], function-based visual field examination [4], and intraocular pressure (IOP) assessment [5]. IOP assessment is usually measured by a tonometer, but a high IOP is not usually a direct diagnosis of glaucoma. Visual field inspection measures the range of a person’s visual field when his sight is focused on the central point. The problem with this diagnostic method is that the level of equipment in each hospital is uneven, and not every hospital has visual field measuring instruments, which makes it Unable to popularize. Therefore, in clinical practice, ophthalmologists generally manually measure the cup-to-disc ratio (CDR) of fundus images for ONH assessment. CDR is the ratio of the vertical optic cup diameter (VCD) to the vertical optic disc diameter (VDD), and a CDR above 0.5 usually indicates a higher risk of glaucoma [6]. As shown in Fig. 1a, b show the complete fundus image of a normal eye and its magnified OD and OC structure, respectively, and Fig. 1c, d show the fundus image glaucoma patient and its magnified OD and OC structure, respectively. However, the manual method of evaluating ONH consumes many labour costs and is not suitable for large-scale screening. In addition, due to the uneven global medical level, manually evaluating ONH is too subjective and too dependent on the clinical experience of doctors. Therefore, there is an urgent need for an automated screening method to assist doctors in diagnosis. At present, many methods for automatically segmenting OD and OC have been proposed, mainly based on model matching methods [7–11] and superpixel based methods [12–15].

Most of these approaches treat the segmentation of OD and OC as two separate problems, ignoring the relationship between OD and OC. In recent years, the success of deep learning (DL) and convolutional neural networks (CNN) in computer vision has led to rapid breakthroughs in the automatic segmentation of OD and OC [16–19]. In fundus image segmentation, the research on OD/OC segmentation is the most complete.Architectures such as Fully Convolutional Network (FCN) [20], U-Net [21], Generative Adversarial Network (GAN) [22], and Region Generative Network (RPN) [23] are used and also tried methods such as multi-scale transformation and polar coordinate transformation. Below we will respectively elaborate on the methods derived from the several above architectures in recent years.

### FCN-based network model

Edupuganti et al. [24] used FCN-8S to segment OD/OC and later improved the network by assigning higher weights to the edges in the loss function. Pohlen et al. [25] proposed a full-resolution residual network (FRRN), using full-resolution residual units (FRRU) to improve the fully convolutional network. Inspired by FRRN, Mohan et al. [26] proposed a Fine-Net with a symmetrical encoder–decoder structure and introduced FRRU. Their subsequent work proposed the P-Net structure [27], which combines DenseBlock and Arous convolution into Dense Arous blocks and cascades Fine-Net to generate high-resolution feature maps. Chen et al. [28] proposed using dilation convolution with different rates to capture multi-scale features and extended it to the Atrous Spatial Pyramid Pooling (ASPP) module. Liu et al. [29] proposed an end-to-end spatially-aware neural network. Spatially dense features are first extracted using Arous CNN, then spatially-aware multi-scale features are obtained using a pyramidal filtering module, and finally, the features are passed to a spatially-aware segmentation module to obtain prediction results.

The above methods all use Arous convolution. Arous convolution can accurately adjust the network receptive field to obtain richer features in the image segmentation task.

### U-Net-based network model

Since U-Net [21] was proposed, it has been widely used in medical image segmentation tasks. It has also inspired researchers to think about U-shaped networks, and many researchers use it as the baseline of segmentation models. Fu et al. [18] proposed M-Net for OD/OC segmentation. They added multi-scale input and output to U-Net and used a multi-label loss function to solve the problem of data imbalance. In addition, they introduced a polar transformation to transform the original fundus image into polar coordinates to improve segmentation accuracy further. Meyer et al. [30] reformulated the segmentation task as a pixel-by-pixel regression task and proposed a pixel-by-pixel multi-task learning method that can combine a U-shaped network model to learn a globally consistent distance distribution across the image and detect the location of both the OD and the central concave. Shah et al. [31] proposed two segmentation models based on parameter sharing branch network (PSBN) and weak region of interest model (WROIM). PSBN includes OD and OC split branches, and the encoder ends of the two split branches share parameters. WROIM consists of two U-shaped networks. First, a small U-shaped network is used to obtain a rough OD area, and then the extracted region of interest (ROI) is input to another U-shaped network for fine segmentation. Gu et al. [32] using pre-trained ResNet blocks as feature encoding blocks, introducing a dense atrous convolution block and residual multi-kernel pooling to capture more high-level features. They applied CE-Net to different 2D medical image segmentation tasks, and all obtained good segmentation performance. Zhang et al. [19] proposed a transferable attention U-net model that uses two discriminators and attention modules to locate and extract invariant features in the dataset, improving the model’s generalization capability. Wang et al. [33] proposed a two-stage neural network that first extracts coarse-level OD regions using a U-shaped prediction network, and then performs fine segmentation using a fine segmentation network that integrates a multi-task learning mechanism and an adversarial learning module. This method improves OD segmentation accuracy, but has less improvement for the more difficult OC segmentation.

Skip connections in U-shaped networks allow features to be passed from the encoder to the decoder to retain some dissipated spatial features and thus improve network performance. However, there may be feature differences between the two sets of merged features, which can still result in the loss of some spatial information. Most of the above U-Net-based methods ignore this problem, which makes it difficult for them to achieve good segmentation performance on OD/OC segmentation.

### GAN-based network model

GAN [22] adopts the idea of gaming, composed of a generator and discriminator, through the confrontation and game between the two to optimise the model to output better results. Wang et al. [34] proposed a patch-based output space adversarial learning framework (POSAL), using MobileNetV2 [35] as the backbone network to extract ROIs and then passing the extracted ROIs to a patch discriminator for adversarial learning. Finally, morphology-aware segmentation loss is proposed to guide the network to generate accurate and smooth results. In subsequent work, they proposed a Boundary and Entropy-Driven Adversarial Learning (BEAL) method [36], consisting of two branches of boundary segmentation and entropy graph segmentation. They introduced adversarial learning to improve the prediction results of both branches.

### RPN-based network model

RPN is derived from Faster-RCNN [23] and is often applied to target detection tasks. Inspired by RPN, Wang et al. [37] used elliptical proposal networks (EPNs) to detect elliptical regions of OD and OC. They used two EPN branches to detect OD and OC separately and added spatial attention between them to guide OC detection. Jiang et al. [38] proposed an end-to-end deep learning framework called JointRCNN. They pass the extracted features to a disc proposal network (DPN) and a cup proposal network (CPN), respectively, and then use a disc attention module to connect the DPN and CPN to determine the location of the OC.

In summary, all methods have contributed significantly to the accurate segmentation of OD and OC. However, most of these segmentation methods ignore the a priori information that the OC is wrapped within the OD. They also deal poorly with the problems of spatial information loss and encoder–decoder feature discrepancies caused by multiple downsampling. Therefore, we propose a residual spatial attention path network (RSAP-Net) to segment OD and OC jointly. The backbone of RSAP-Net is a U-shaped convolutional network. We replace the skip connections of the U-shaped network with a residual path with spatial attention (RSAP), which consists of a residual convolutional block and a spatial attention block to mitigate feature differences between encoders–decoders and aggregate global contextual information. Considering that the partitioning of OC is more difficult than the partitioning of OD, in most cases the partitioning of OC is difficult to define limits [39]. To improve the model segmentation performance further, we also designed a preprocessing data method combining a multi-scale Retinex colour restoration algorithm (MSRCR) [40] and a polar transformation (PT), called MSRCR-PT. M-Net’s use of PT inspired us [18]. After carefully studying the characteristics of PT, we found that since PT is a pixel-direction mapping, the data enhancement on the original fundus image is equivalent to the data enhancement in polar coordinates. Therefore, we first used the MSRCR algorithm for the original fundus image’s colour restoration and then processed the enhanced image for polar coordinate transformation based on the equivalent enhancement characteristics. This fusion of MSRCR and PT pre-processing can enhance the OD and OC edge parts while equalising the CDR, thus improving the segmentation performance of the model.

The main contributions of this article are as follows:

1. We propose a residual spatial attention path network for automatic joint segmentation of OD and OC, named RSAP-Net.
2. We propose a new residual path with spatial attention (RSAP) to replace skip connections in conventional U-shaped networks to mitigate feature differences between encoders–decoders while also improving the segmentation performance of OD and OC. We verified the effectiveness of RSAP through ablation experiments.
3. Given the a priori condition that OC is always wrapped in OD, the boundary segmentation of OC is more challenging. For this reason, we designed a data preprocessing method, MSRCR-PT, that integrates MSRCR and PT, which can enhance the edge of OD and OC while equalizing CDR to improve model segmentation performance further. We verified the effectiveness of MSRCR-PT through ablation experiments.
4. Finally, we evaluated our method on the Drishti-GS1 dataset. Compared with existing methods, this method has achieved outstanding segmentation results. In the segmentation effect of OD and OC, F1 is 0.9752 and 0.9012, respectively. BLE is 6.63 pixels and 11.97 pixels, respectively.

## Results

In this part, we will describe the data sets used in the experiment, the experimental settings, and the evaluation indicators used in this article. At the end of this part, we designed a large number of comparative experiments to evaluate the segmentation performance of the model.

### Data preparation

#### Dataset

The DRISHTI-GS1 standard dataset [41] has 101 full eye fundus images, including 50 training images and 51 test images, including 70 glaucomatous lesions and 31 images of normal eyes.

**Fig. 2:**
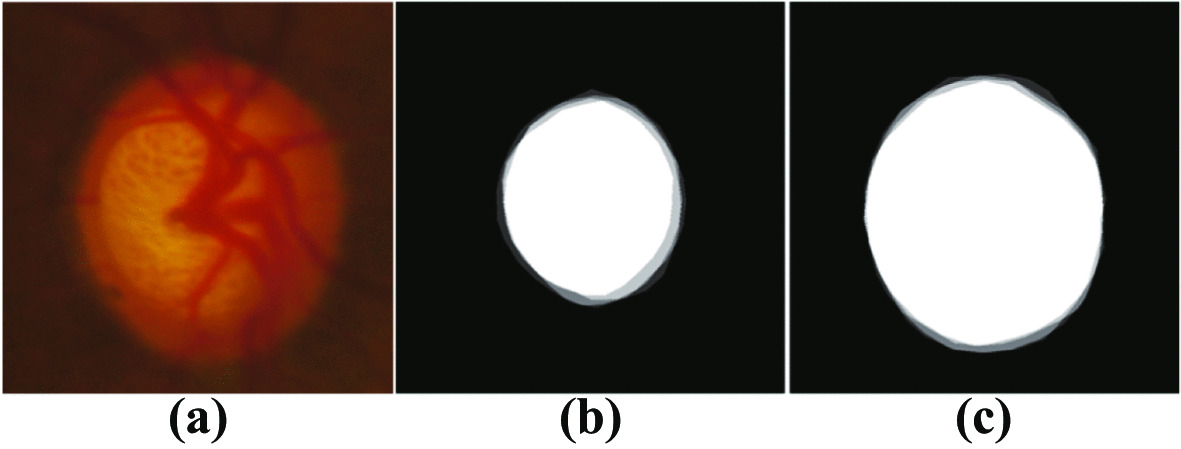
**a** Retinal fundus image, **b** OC area labelled by four experts, **c** OD area labelled by four experts

The original images for the dataset were provided by Aravind eye hospital, Madurai, who selected an approximately equal number of men and women, aged 40–80 years, with glaucoma and non-glaucoma patients for fundus image acquisition. All images were acquired with dilated pupils and captured according to the following data collection protocol: OD-centred High-resolution fundus images of $2896 \times 1944$ pixels were acquired with a field of view of 30°. Finally, by removing the surrounding non-fundus black area, the image area with the retinal structure is extracted from the original image, thereby obtaining a fundus image with a resolution of about 2047 $\times$ 1760. As shown in Fig. 2, each image was manually labelled by four glaucoma specialists with 3, 5, 9 and 20 years of experience, respectively.

#### Data augmentation

For the problem of the small number of images in the data set, we first use the YOLOv2 [42] model to extract OD images and then intercept images of different sizes according to the OD centre point, including 400 $\times$ 400, 500 $\times$ 500, 550 $\times$ 550, 600 $\times$ 600, 650 $\times$ 650, 700 $\times$ 700, 750 $\times$ 750, 800 $\times$ 800, 850 $\times$ 850, 900 $\times$ 900. Then the fundus image is randomly flipped horizontally, vertically, and rotated within the range of [0° 360°]. Before training the network, the size of the input image is scaled to the standard 512 $\times$ 512. This method can expand the number of training samples and increase the diversity of training samples.

### Experimental setup

As shown in Table 1, all training and testing for this work were carried out in the following hardware environment: CPU type Intel^®^ Xeon^®^ Glod 5218 Processor 2.30 GHz, running memory 187G, GPU type NIVIDA Quadro RTX 6000 and video memory size 24G. The operating system used for the experiments was Linux Ubuntu 16.04. We use the Pytorch [43] deep learning framework to implement RSAP-Net, the programming language used to build the network model was Python 3.7. The main library packages used in the programming process were Pytorch 1.8, openCV 4.1.2, Numpy 1.18.1, etc. During the training period, the learning rate was set to 0.001, the weight decay coefficient to 0.0005, the momentum to 0.9 and the Stochastic gradient descent (SGD) algorithm were used as the optimiser of the network. The training is iterated for a total of 400 epochs, and the output segmented image size is 512 $\times$ 512.

**Table 1:** Experimental hardware and software information

Category	Hard/software environment
CPU	Inte^®^Xeonl®^®^Glod 5218 Processor 2.30 GHz
GPU	NIVIDA Quadro RTX 6000 Video memory 24G
RAM	187G
OS	Linux Ubuntu 16.04
Frame	Pytorch1.8 openCV4.1.2 Numpy1.18.1
Language	Python3.7

### Evaluation metrics

In order to quantify the experimental results, we evaluate the performance of the different methods, mainly using F1 and boundary distance localization error (BLE), which are widely used by the research community. In the ablation experiments, we also compared specificity (SPC), sensitivity (SEN) and accuracy (ACC). Among them, the definition of F1 is:

1 $$\begin{aligned} F1=2\times \frac{\text {Precision}\times \text {Recall}}{\text {Precision}+\text{ Recall }} \end{aligned}$$

Precision and Recall are defined as:

2 $$\begin{aligned} \text{ Precision }=\frac{\mathrm {TP}}{\mathrm {TP}+\mathrm {FP}} \end{aligned}$$

3 $$\begin{aligned} \text{ Recall }=\frac{\mathrm {TP}}{\mathrm {TP}+\mathrm {FN}} \end{aligned}$$

The definitions of SPC, SEN and ACC are:

4 $$\begin{aligned} \text {SPC}=\frac{\mathrm {TN}}{\mathrm {N}} \end{aligned}$$

5 $$\begin{aligned} \text {SEN}=\frac{\mathrm {TP}}{\mathrm {P}} \end{aligned}$$

6 $$\begin{aligned} \text {ACC}=\mathrm {SPC} \times \frac{\mathrm {N}}{(\mathrm {P}+\mathrm {N})}+\mathrm {SEN} \times \frac{\mathrm {P}}{(\mathrm {P}+\mathrm {N})} \end{aligned}$$

In the above formula, TP, TN, FP and FN represent true-positive, true-negative, false-positive and false-negative cases, respectively, while P and N represent positive and negative samples. Since BLE can better represent the segmentation effect of the boundary, this paper introduces BLE to evaluate the boundary distance (in pixels) between the edge $C_{0}$ of the model segmentation result and the edge $C_{g}$ of the labelling result. The definition of BLE is:

7 $$\begin{aligned} \mathrm {BLE}\left( \mathrm {C}_{0}, \mathrm {C}_{\mathrm {g}}\right) =\frac{1}{\mathrm {~N}} \sum _{\theta =0}^{N-1} \sqrt{\left( \mathrm {d}_{\mathrm {g}}^{\theta }\right) ^{2}-\left( \mathrm {d}_{0}^{\theta }\right) ^{2}} \end{aligned}$$

where $d_{g}^{\theta }$ and $d_{0}^{\theta }$ represent the Euclidean distance from the centre point of OD in the $\theta$ direction to $C_{g}$ and $C_{0}$, and 24 equidistant points ($N=24$) are set in the evaluation. The smaller the BLE, the better the segmentation effect. BLE is smaller means better segmentation performance.

### Model performance improvement

A simple encoder–decoder network and an MBCE module are used to form the baseline network. First, to verify the effectiveness of the MBCE module, we set up ablation experiments to demonstrate the efficacy of MBCE, and the experimental results are shown in Table 2.

**Table 2:** MBCE ablation experiment results on the Drishti-GS1 dataset

Method	F1(mean/std)	
	OD	OC
Encoder–decoder	0.9517/0.018	0.8531/0.11
Baseline (encoder–decoder+MBCE)	0.9541/0.062	0.8678/0.13

In order to verify the effectiveness of the MSRCR-PT method and the RSAP module, we designed a set of ablation experiments and evaluated them on the Drishti-GS1 dataset. The combination method is as follows:

1. Baseline: consists of an encoder, a contextual feature extraction module and a decoder. The input is the original fundus image, and the loss function is the cross-entropy loss function.
2. Baseline+PT: On the basis of (1), use polar transformation on the original fundus image.
3. Baseline+MSRCR-PT: On the basis of (1), the original fundus image is first processed with the MSRCR algorithm, and then the processed image is subjected to polar coordinate transformation.
4. Baseline+RSAP: The network structure is shown in Fig. 5a, based on (1) , the residual spatial attention path module is added between the encoder–decoder.
5. Baseline+RSAP+MSRCR-PT: On the basis of (4), the original fundus image is first processed with the MSRCR-PT method, and then the processed image is used as input to the model.

**Table 3:** RSAP-Net ablation experiment results on the Drishti-GS1 dataset

Method	Parament (KB)	OD				OC			
		F1(mean/std)	ACC	SPC	SEC	F1(mean/std)	ACC	SPC	SEC
Baseline	77081	0.9541/0.062	0.9655	0.968	0.8249	0.8678/0.13	0.9689	0.9694	0.8768
Baseline+PT	77081	0.9649/0.049	0.9683	0.9701	0.8266	0.8764/0.11	0.9712	0.9735	0.8794
Baseline+MSRCR-PT	77081	0.9668/0.026	0.9756	0.9778	0.8567	0.8803/0.09	0.9766	0.9781	0.9036
RSAP-Net (Basline+RSAP)	89659	0.9633/0.031	0.9764	0.9775	0.8458	0.8742/0.09	0.9772	0.9788	0.8959
RSAP-Net+MSRCR-PT	89659	0.9752/0.012	0.977	0.9781	0.8564	0.9012/0.08	0.9775	0.9796	0.9154

The experimental results are shown in Table 3. It can be seen that both MSRCR-PT and RSAP can effectively improve the segmentation performance of the model. When Baseline+MSRCR-PT is applied, since the MSRCR algorithm adaptively enhances the original fundus image, and the polar transformation helps balance the cup-to-disk ratio, the segmentation performance of the model has been significantly improved. The performance of OD and OC in F1 is 1.27% and 1.25% higher than the Baseline, respectively. When Baseline+RSAP+MSRCR-PT is applied, RSAP helps the model retain more spatial feature information while aggregating global context information, which further improves the segmentation performance of the model. The performance of OD and OC in F1 is 2.11% and 3.34% higher than Baseline respectively.

**Fig. 3:**
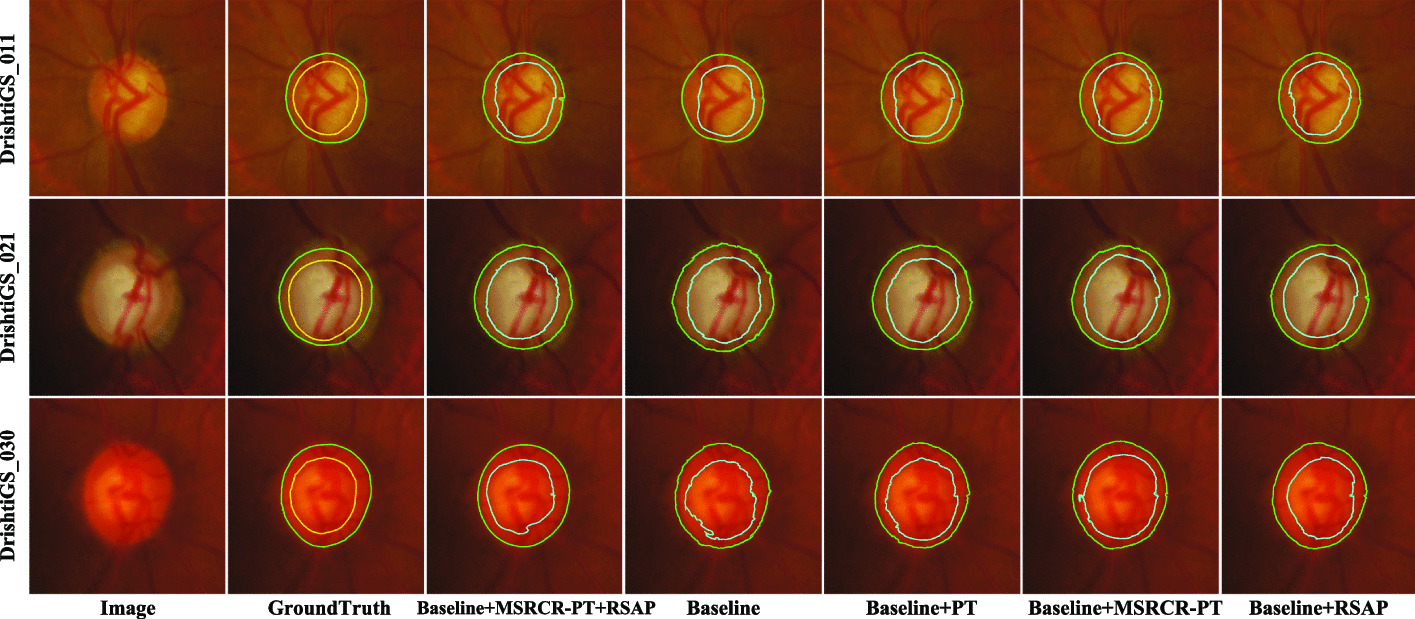
A visual example of RSAP-Net ablation experiment results on the Drishti-GS dataset

Figure 3 shows a visualisation example of RSAP-Net’s ablation experiment results on the Drishti-GS dataset. It can be seen that compared with Baseline, the use of MSRCR-PT significantly improves the accuracy of OD and OC segmentation, making the edge of the segmentation result more smooth. The segmentation results achieved using both MSRCR-PT and RSAP are also the closest to GroundTruth, proving that both methods help the model segment the boundaries of OD and OC, eliminate noise and reduce the negative impact of background.

### Compare with state-of-the-art methods

To demonstrate the segmentation performance of RSAP-Net, we compared the experimental results with those of FCN [20], U-Net [21], M-Net [18], POSAL [34], JointRCNN [38], CE-Net [32], Stack-U-Net [44], BGA-Net [45] and DCGAN‘[46]. All experiments were evaluated on the Drishti-GS1 [41] dataset.The comparison results are shown in Table 4. Fu et al. [18] uses U-Net as the Baseline, uses polar transformation to balance the ratio between OD, OC and background, and adds multiple input and output operations to the model. This method can obtain multi-scale feature information, but due to the main body’s relatively simple structure, the model’s segmentation performance is relatively low. The F1 scores of the optic disc and optic cup are only 0.959 and 0.866. The POASL [34] framework enhances the robustness of the deep network in order to cope with the domain drift problem, ignoring the spatial overlap relationship between OD and OC, and is unable to segment the OC boundary accurately, which also leads to its lack of segmentation accuracy. JointRCNN [38] treats the OD and OC segmentation problem as a target detection problem, using an optic disc attention module to connect two improved fast-RCNNs: DPN and CPN. Although this method achieves joint OD and OC segmentation, it is susceptible to fundus vascular interference and does not segment boundary contours well. CE-Net [32] uses pre-trained ResNet-34 as the coding block of the network, adds a new context extraction module to capture more high-level features, and proves its effectiveness in other two-dimensional medical image segmentation tasks. However, the segmentation effect for OD and OC is not good, and the F1 scores of OD and OC are only 0.968 and 0.869. The main body of Stack-U-Net [44] is two stacked U-Net, which increases the model complexity, but the performance improvement is not significant. The BGA-Net [45] consists of a segmentation backbone, a boundary auxiliary branch and two adversarial networks. Although there is a good improvement in segmentation performance for both OD and OC, the number of parameters grows linearly as the model becomes more complex, significantly increasing the computational cost. DCGAN [46] combined DCNN and GAN to propose a DCGAN-based model to jointly segment OC and OD. DCGAN achieved good segmentation performance on the segmentation of OD, but performed poorly on the segmentation accuracy of OC, with an F1 score of only 0.8631.

**Table 4:** Comparison of quantitative results of different methods on the Drishti-GS1 dataset

Method	Year	OD (mean/std)		OC (mean/std)	
		F1	BLE(px)	F1	BLE (px)
FCN [20]	2014	0.9321/0.102	8.90/5.74	0.8170/0.103	21.83/15.67
U-Net [21]	2015	0.9600/0.020	7.23/4.51	0.8500/0.100	19.53/13.98
M-Net [18]	2018	0.9590/0.040	7.97/8.29	0.866/0.110	17.05/12.76
Stack-U-Net [44]	2018	0.9700/0.020	6.47/3.51	0.8900/0.090	14.39/7.18
POSAL [34]	2019	0.9650/–	–/–	0.8580/–	–/–
CE-Net [32]	2019	0.9688/0.003	5.04/3.69	0.8699/0.117	16.06/13.11
JointRCNN [38]	2020	0.9640/–	–/–	0.8640/–	–/–
BGA-Net [45]	2021	0.9750/0.019	7.01/3.53	0.8980/0.08	14.37/9.29
DCGAN [46]	2022	0.9746/0.017	7.35/4.83	0.8631/0.113	18.69/13.48
Proposed method	–	0.9752/0.012	6.33/3.30	0.9012/0.08	11.97/6.12

**Fig. 4:**
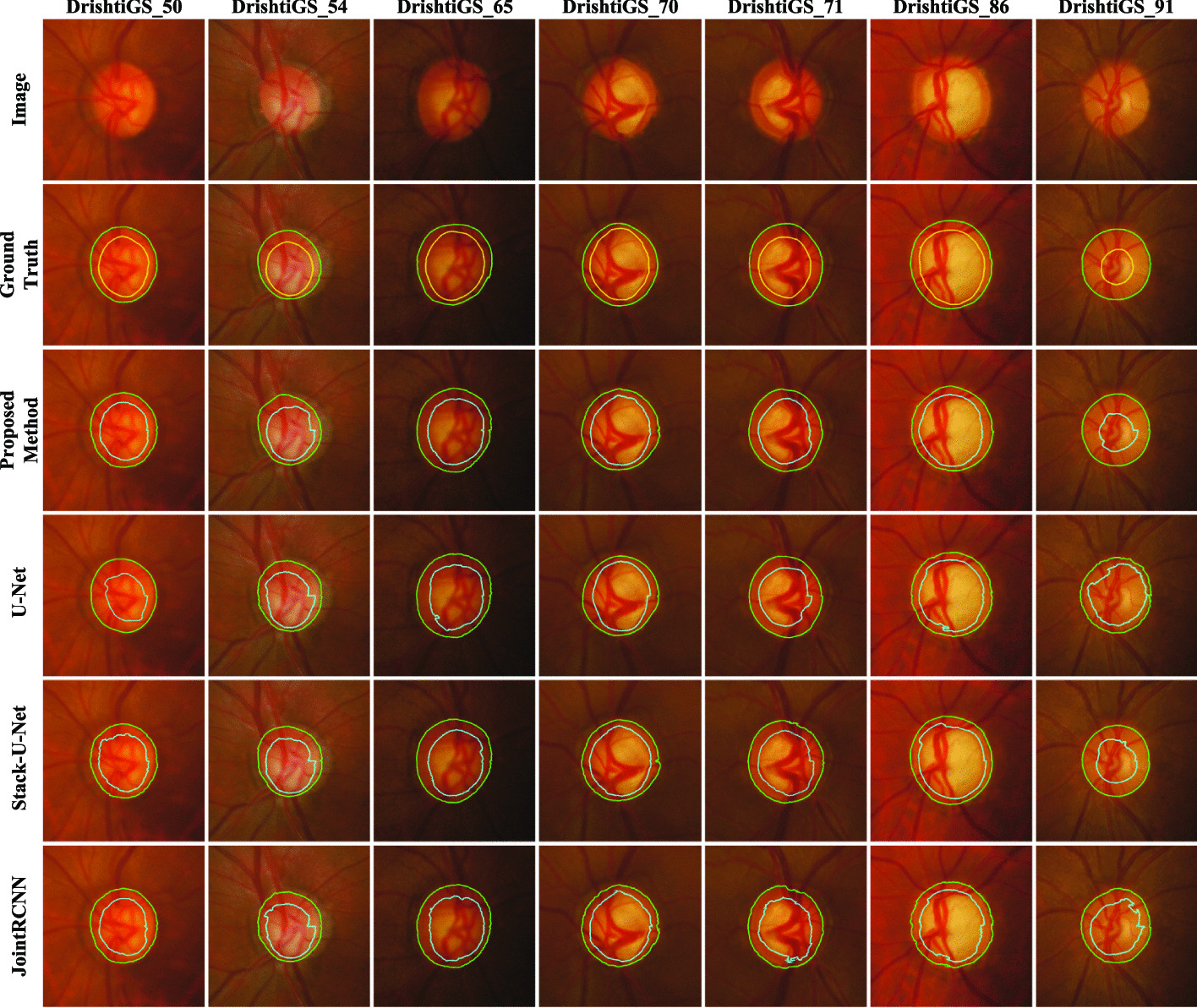
Examples of segmentation results of different methods on the Drishti-GS1 dataset

The deep convolutional neural network model RSAP-Net proposed in this paper performs joint segmentation of OD and OC and cooperates with the MSRCR-PT preprocessing method to achieve excellent segmentation performance on the Drishti-GS1 data set. Especially in OC segmentation, the F1 score and BLE value of RSAP-Net reached 90.12 and 11.97, respectively, which are 0.32% and 2.4px higher than the most advanced BGA-Net. Figure 4 shows the OD and OC segmentation edge profiles of RSAP-Net with U-Net, Stack-U-Net and JointRCNN on seven samples of the Drishti-GS1 dataset. Comparison of the segmentation effect plots of the four methods shows that the proposed RSAP-Net model segments the OD and OC boundaries better than the other methods. The error between the edges of the segmented region and the edges of GroundTruth is the smallest, especially in OC segmentation.

## Discussion

1. *Running time* The entire training phase of RSAP-Net takes approximately 4.5 h on a single NIVIDA Quadro RTX 6000 (400 epochs). When tested, generating the final segmentation map for a fundus image took only 1.32 s, which is faster than existing methods such as M-Net [18], which takes 1.83s and Stack-U-Net [44], which takes 2.21 s.
2. *Is the MSRCR-PT method effective when used with other models?* To further demonstrate the effectiveness of the MSRCR-PT method, we selected the classical U-Net [21] model for the ablation experiments, and the results are shown in Table 5. It can be seen that when using the U-Net + MSRCR-PT method, OD and OC are 0.81% and 2.93% higher than U-Net in F1, and 0.85 px and 6.42 px lower in BLE, respectively, where the segmentation performance improvement of OC is more obvious.
3. *Limitations and prospects* First of all, in this work, we only studied and analysed the OD and OC segmentation tasks, and although we achieved some results, this does not show the effectiveness of our method for other segmentation tasks. Therefore, it is natural to question whether the MSRCR-PT preprocessing method and RSAP can be generalised to other tasks. We have found in our extensive experiments that the MSRCR algorithm also has a positive effect on the adaptive enhancement of fundus vessels, which will be the next step in our work.

**Table 5:** U-Net uses PT and MSRCR-PT ablation experiments on the Drishti-GS1 dataset

Method	Parament (KB)	OD (mean/std)		OC (mean/std)	
		F1	BLE(px)	F1	BLE(px)
U-Net[21]	67535	0.9600/0.020	7.23/4.51	0.8500/0.100	19.53/13.98
U-Net+PT	67535	0.9658/0.014	6.85/3.48	0.8681/0.110	15.63/8.51
U-Net+MSRCR-PT	67535	0.9681/0.012	6.38/3.43	0.8793/0.090	13.11/6.87

## Conclusions

In order to alleviate the problem of feature differences between codecs in end-to-end networks, this paper proposes a codec network connected by a residual spatial attention path to achieve joint segmentation of OD and OC. RSAP-Net is composed of a residual encoder, multi-branch context extraction module, decoder and RSAP. On the one hand, RSAP effectively alleviates the problem of feature differences between encoders and decoders. On the other hand, the multi-branch context feature extraction module can capture more high-level features and retain more spatial information. Before training, considering the positional overlap between OD and OC in the fundus image, the MSRCR-PT pre-processing method is used to process the original fundus image. MSRCR-PT combines a multi-scale colour restoration algorithm and a polar coordinate transformation method. While adaptively enhancing the fundus image, it can also balance the cup-to-disk ratio to prevent over-fitting during model training. Finally, we proved the effectiveness of the model through ablation experiments. Compared with the existing methods, RSAP-Net achieved better segmentation results on the Drishti-GS1 dataset.

## Methods

**Fig. 5:**
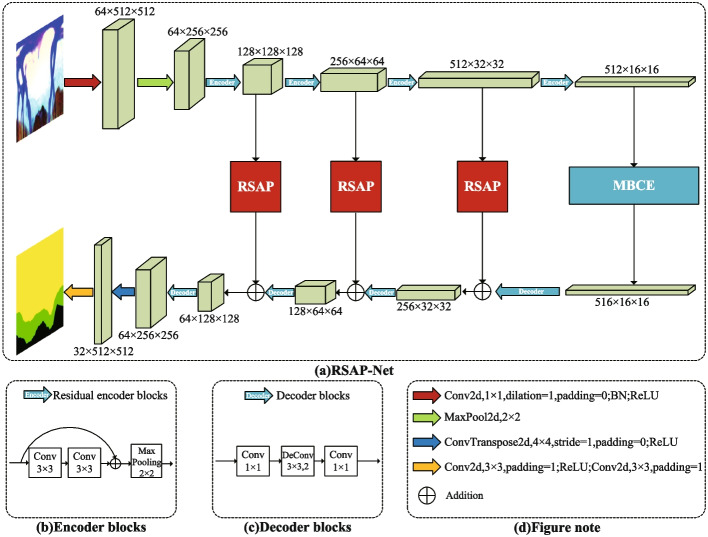
**a** RSAP-Net overall network architecture, **b** Encoder module, **c** Decoder module, **d** figure note

The overall framework of RSAP-Net is shown in Fig. 5. We choose a U-shaped network as the backbone of RSAP-Net, which consists of four parts: an encoder part, a multi-branch context extraction module, a residual spatial attention path (RSAP) and a decoder part. In the encoder part, the initial input scale of the original image is 512 $\times$ 512. After five times of down-sampling operations, five different scale feature maps of 256 $\times$ 256, 128 $\times$ 128, 64 $\times$ 64, 32 $\times$ 32, and 16 $\times$ 16 are obtained. As shown in Fig. 5a, the feature encoder module uses four residual blocks as the backbone. It then adds maximum pooling layer for downsampling, reducing the upper layers’ computational complexity while preventing overfitting. The multi-branch context extraction module consists of a multi-branch dilated convolution block (MDB) and a global information coding block (GIC). The decoder part comprises four decoding blocks and uses deconvolution to perform upsampling to obtain refined edges and reconstruct depth features. Each decoding block consists of two 1 $\times$ 1 convolutional layers and a 3 $\times$ 3 deconvolutional layer with a step size of 2. Between the encoder and decoder, we have added three RSAP modules, using RSAP as an auxiliary path to transfer the characteristic information generated by the shallower layers of the network, especially the spatial information of OD and OC. RSAP can filter noise in the feature map and resolve boundary-blurring caused by multilayer downsampling and upsampling, as well as mitigate feature differences between the coder-decoder and enhance the edge features of OD and OC, which are essential for OD and OC segmentation.

### Residual spatial attention path module

In the encoder–decoder network for OD and OC segmentation, continuous down-sampling and up-sampling will lose much characteristic information and cause boundary blur problems. As the number of network layers increases, this problem will become more serious. U-Net [21] proposes a skip connection to retain as much of the lost feature information as possible. Although the skip connection bridges the encoding block with the decoding block and preserves some of the feature information. However, the features obtained by the decoder after intense computation are higher-level features. In contrast, the features obtained by the encoder through the shallower network calculations belong to low-level features. Therefore, simply bridging the coder-decoder via a skip connection cannot eliminate the difference in features between the two. The deeper the skip connection, the smaller the difference because the deeper the encoder goes, the deeper the computation, which gradually reduces the computational difference with the decoder. Conversely, the difference in features is greater at the shallower levels of the network.

**Fig. 6:**
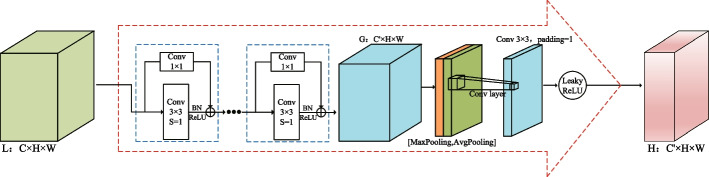
Residual spatial attention path (RSAP module)

To further alleviate the feature differences between encoders–decoders and the problem of boundary ambiguity. we have added three Residual spatial attention paths between codecs from top to bottom. These residual paths are composed of several residual convolution blocks and a redesigned spatial attention mechanism. The residual convolution block is used to simulate the residual convolution operation in the encoder. The spatial attention mechanism is used to focus on the “where” feature is more meaningful, and the spatial domain information in the input feature map is processed accordingly. Improve accuracy while reducing the number of calculations. Let the network learn the edge characteristics of OD and OC more effectively.

As shown in Fig. 6, the input to RSAP is the low-level feature map $L \in R^{C \times W \times H}$ output from the encoder side. The higher-level feature map $G \in R^{C^{\prime } \times W \times H}$ is output using the residual block to simulate the residual convolution operation in the encoder. The channel information of the feature map is then aggregated by using the maximum pooling and average pooling operations to generate two 2D maps: $X_{\max }^{s} \in R^{1 \times W \times H}$ and $X_{a v g}^{s} \in R^{1 \times W \times H}$, representing the global maximum pooling feature and the global average pooling feature, respectively. Finally, the higher-level feature map $H \in R^{C^{\prime } \times W \times H}$ generated by RSAP is summed with the corresponding higher-level feature map output from the decoder side as the input to the next decoding block. In short, the spatial attention mechanism in RSAP is computed as:

8 $$\begin{aligned} {\text{F}}_{{\text{s}}} ({\text{X}}) & = {\text{ LR }}\left( {{\text{C}}^{{3 \times 3}} ({\text{Concat}}({\text{Max}}({\text{X}}),{\text{Avg}}({\text{X}})))} \right) \\ & = {\text{LR}}\left( {{\text{C}}^{{3 \times 3}} \left( {{\text{Concat}}\left( {X_{{\max ^{{\text{s}}} }} ,X_{{{\text{avg}}}}^{{\text{s}}} } \right)} \right)} \right. \\ \end{aligned}$$

Here, LR represents the LeakyReLU activation function,Max means maximum pooling,Avg means average pooling, Concat means concatenate, and C$^{3 \times 3}$ represents the convolution operation with a convolution kernel size of 3 $\times$ 3.

### Multi-branch context extraction module

Gu et al. [32] Thinking about the context extraction module inspired us. At the bottom of the backbone network, we introduced the CE-Net context extractor and adjusted it for OD and OC segmentation tasks. The adjusted module is called the multi-branch context feature extraction module, composed of a multi-branch dilated convolution block (MDC) and a global information coding block (GIC). This module is primarily used to extract contextual semantic information and generate high-level feature maps.

**Fig. 7:**
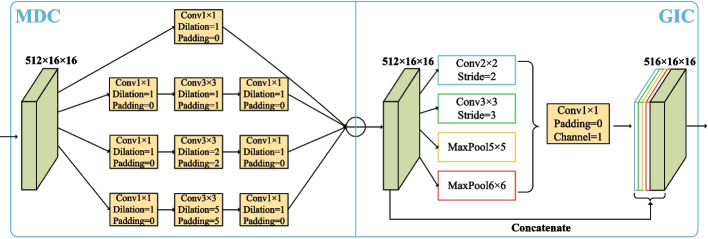
Multi-branch context extraction module. It is composed of a multi-branch dilated convolution block and a global information coding block

#### MDC: multi-branch dilated convolution block

Aiming at the problem that multiple downsampling in neural networks will reduce image resolution and lose feature information, [47] proposed dilated convolution. They introduced a hyperparameter called “dilation rate” to the convolutional layer to define the spacing between values when the convolution kernel processes data. Using dilation convolutions with different dilation rates allows ordinary convolutions to have a larger receptive field with the same parameters and calculations.

The output resolution calculation formula of dilated convolution is as follows:

9 $$\begin{aligned} W_{\text{ out } }=\frac{W_{\text{ in } }+2 p-d(k-1)-1}{s}+1 \end{aligned}$$

Among them, $W_{in}$,$W_{out}$ are the input image’s width and the output image’s width, p is the padding, d is the dilation rate, k is the size of the convolution kernel, and s is the step size. Inspired by dilated convolution, we merged the Inception block [48] and dilated convolution and introduced MDC block to encode deep feature maps. As shown in Fig. 7, the MDC is divided into four cascade branches. We use three convolutions of 1, 2 and 5 dilation rates to expand the receptive field and add a $1 \times 1$ convolution in each branch to correct linear activation. By applying dilated convolution with different dilation rates, MDC blocks can extract richer contextual features.

#### GIC: global information coding block

In order to deal with the problem of different target sizes in OD and OC segmentation tasks, we introduced a global information coding block(GIC). As shown in Fig. 7, GIC consists of two convolution branches and two pooling branches. The convolution branches are convolution kernels with $2 \times 2$ steps of 2 and $3 \times 3$ steps of 3. The pooling branches are two maximum pooling operations of 5 $\times$ 5 and 6 $\times$ 6. GIC can achieve four effective fields of view of different sizes to detect targets of different sizes. In order to reduce the number of parameters and reduce the computational cost, we use $1 \times 1$ convolution. Finally, the low-level feature maps whose channels are equal to 1 output by the four branches are up-sampled to make them reach the same size as the original feature maps and concatenated.

### MSRCR-PT: multi-scale Retinex color restoration and Polar transformation

Before inputting the image into the convolutional neural network model for training and testing, it is necessary to design an effective pre-processing method, and the pre-processing method designed in this paper focuses on the following two key points:

1. The boundary between OD and OC is blurred, especially the boundary of OC.
2. OC is too small in the image, which is very easy to cause overfitting during training.

#### The significance of fundus retinal image pre-processing

**Fig. 8:**
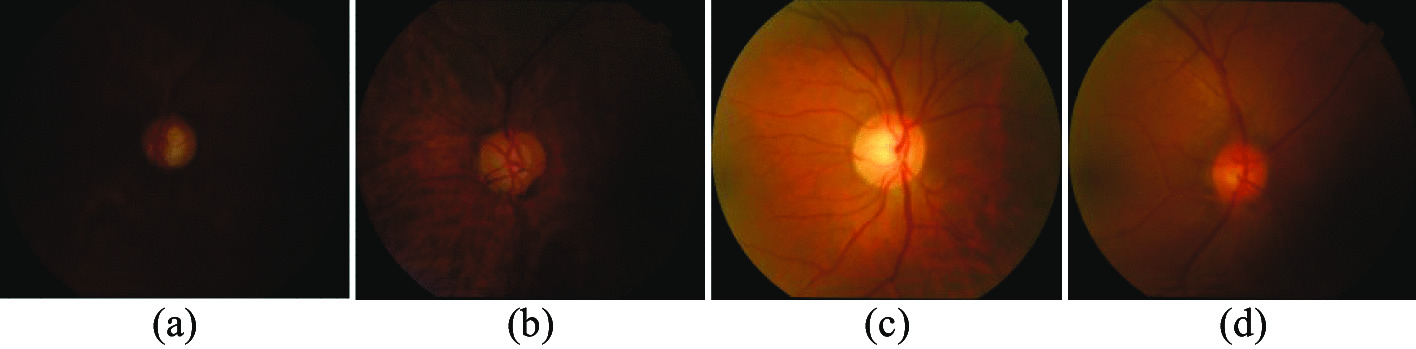
Comparison of Drishti-GS1 standard fundus retinal data sets for light and dark and illumination conditions

The acquisition of fundus retinal images is often affected by several factors, such as uneven illumination during acquisition, different shapes of lesions and fundus colours from patient to patient, and technical problems with the acquisition equipment, all of which can lead to uneven quality of the acquired images. Figure 8 shows the original fundus retinal images for each of the four patients from the Drishti-GS1 dataset. It is clear that there is a large difference between the light and darkness in patients (a, b) and (c, d), and that the image in (d) is itself unevenly illuminated, with the lower right corner being significantly darker.

**Fig. 9:**
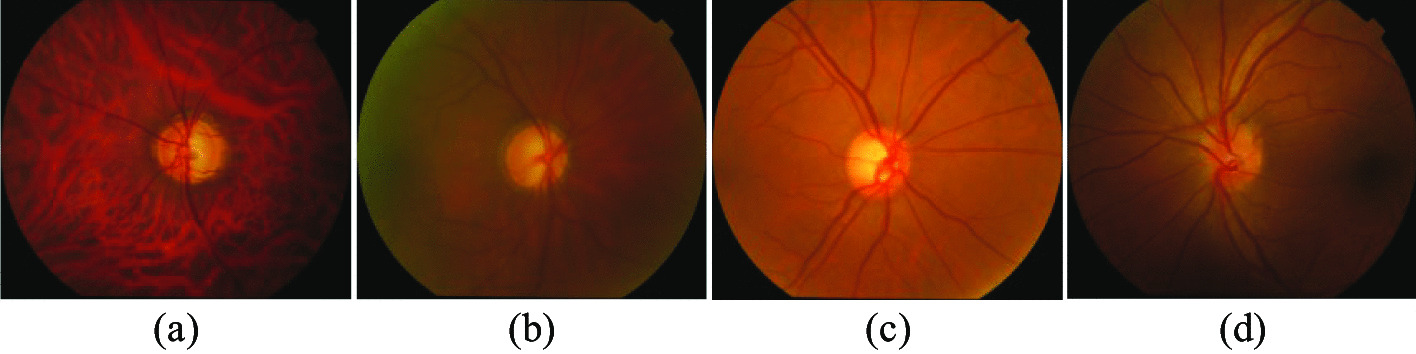
Drishti-GS1 standard fundus retinal dataset colour comparison

Figure 9 show the original fundus retinal images of four additional patients from the Drishti-GS1 dataset. It is clear that the colour of the fundus images of these four patients is very different, with (a) the darkest image and (b) (c) (d) the lighter image, and the overall fundus image being reddish in colour. In the optic disc-optic cup segmentation task, all of these factors make it more difficult to segment the boundaries between the optic disc and the optic cup, and between the optic disc-optic cup and the background.

**Fig. 10:**
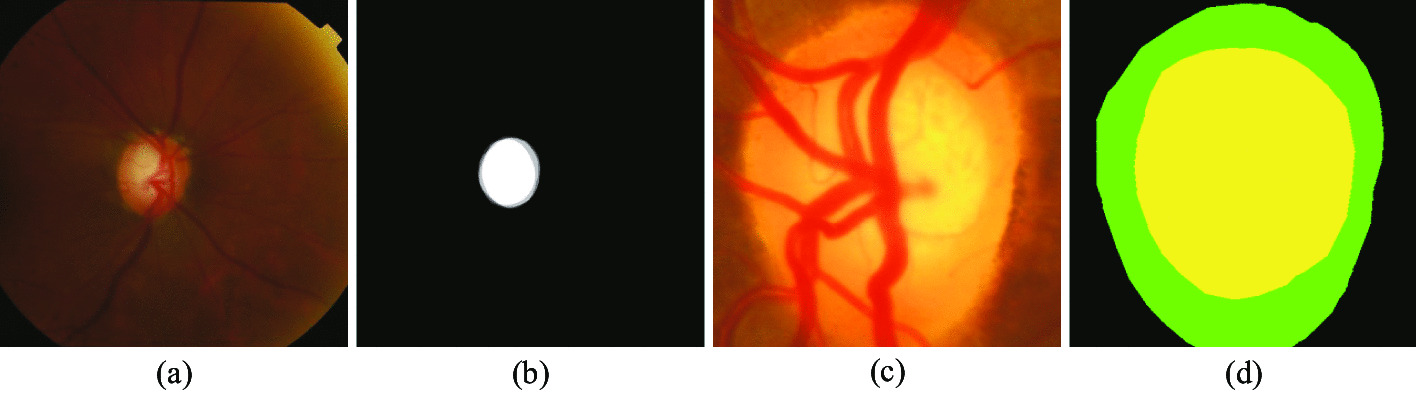
**a** Original image; **b** ground truth (optic cup portion); **c** enlarged ROI; **d** enlarged ground truth (were yellow, green, and black areas indicate the optic cup, optic disc, and background, respectively)

The key point in the optic disc optic cup segmentation task is to accurately segment the OD and OC from the fundus image. The difficulty lies in the boundary segmentation between OD and OC and the boundary segmentation between OD and background. Figure 10 shows the original unpreprocessed fundus image and Fig. 10b shows the gold standard for segmentation of the optic cup in the original fundus image. The optic cup occupies too few pixels in the whole image compared to the rest of the image, which is very easy to overfit in the training of a deep convolutional neural network. Figure 10c, d show the magnified original fundus retinal image and the ground truth respectively. By comparing (c) and (d) it is clear that the boundary between the optic cup and the optic disc is very blurred, which very much affects the model’s ability to accurately segment the optic cup boundary.

#### Pre-processing method: MSRCR-PT

**Fig. 11:**
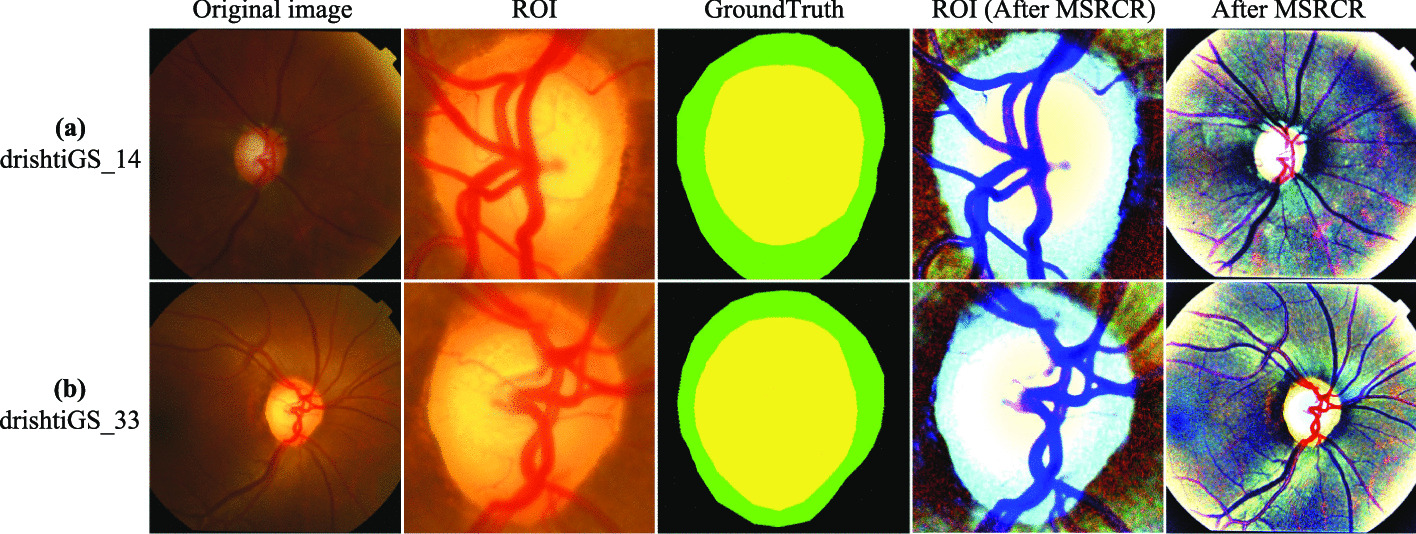
Comparison of fundus images before and after processing by the MSRCR algorithm. Where the yellow, green and black areas indicate OC, OD and background, respectively

M-Net [18] uses a polar transformation (PT) to present better the spatial overlap relationship between OD and OC to the neural network model. A closer look at the properties of PT reveals that because PT is a mapping of pixel directions, data enhancement on the original fundus image is equivalent to data enhancement in polar coordinates. In order to improve the performance of OD segmentation while still coping with the complex problem of OC segmentation, we have designed a pre-processing method that incorporates the MSRCR algorithm and PT. Before performing polar transformation on the fundus image, we first colour-enhanced the original fundus image using the MSRCR method. Retinex, a combination of the words Retina and Cortex, is a commonly used image enhancement method. It was proposed by Edwin. H. Land in 1963 [41]. It can balance three aspects: dynamic range compression, edge enhancement and colour constancy, thus allowing adaptive enhancement of a wide variety of different types of images. After more than 40 years of development, the Retinex algorithm has gradually improved from the single-scale Retinex algorithm (SSR) to the multi-scale Retinex algorithm (MSR), and then to the multi-scale Retinex colour restoration algorithm (MSRCR). After carefully observing the Drishti-GS1 data set, we found that the OD and OC edges of many fundus images are relatively blurred, especially the OC part. In order to show the boundary between OD and OC more clearly, we apply the MSRCR algorithm to the segmentation of OD and OC. The steps of the MSRCR algorithm are as follows Algorithm 1:
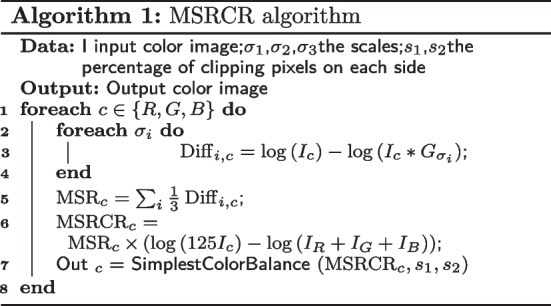


Figure 11 shows the effect of the fundus image after processing by the MSRCR algorithm. The OD and OC edges are much clearer after processing than the original fundus image, especially the OC border.

**Fig. 12:**
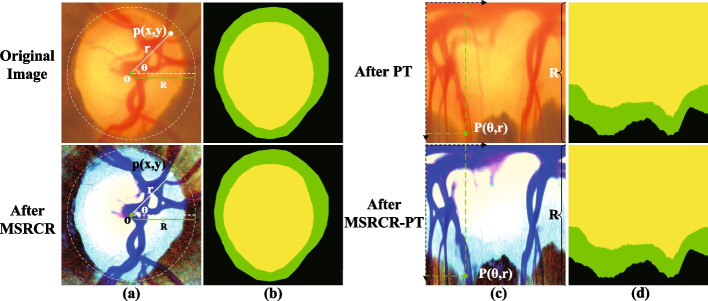
**a** Fundus image in the right-angle coordinate system, **b** GroundTruth in right-angle coordinate system, **c** Fundus image in polar coordinate system, **d** GroundTruth in polar coordinate system. where the yellow, green and black areas indicate OC, OD and background, respectively

Finally, we converted the fundus images processed by the MSRCR algorithm to a polar coordinate system on a pixel-by-pixel basis to equalize the cup-to-disc ratio and further improve the segmentation performance of OD and OC. As shown in Fig. 12a, the point $p=(x, y)$ represents the point on the image plane of the fundus, corresponding to the point $P(\theta , r)$ on the polar coordinate system, where r and $\theta$ are the radius and directional angle of the origin p, respectively. Figure 10 visualises the effectiveness of the MSRCR-PT method for OD and OC segmentation. When comparing the fundus images processed with GroundTruth using only PT and using the MSRCR-PT method, it is clear that the OD and OC boundaries are much clearer in the fundus images processed with MSRCR-PT.

The transformation relationship between polar coordinates and Cartesian coordinates is as follows:

10 $$\begin{aligned} \left\{ \begin{array} { l } { x = r {\text { c o s }} \theta } \\ { y = r {\text { s i n }} \theta } \end{array} \leftrightarrow \left\{ \begin{array}{c} r=\sqrt{x^{2}+y^{2}} \\ \theta =\tan ^{-1} y / x \end{array}\right. \right. .\end{aligned}$$

### Loss function

The cross-entropy loss function is very effective for multi-classification networks. Since OD and OC segmentation needs to segment the three categories of background, OD and OC, we use the cross-entropy loss function as the loss function of RSAP-Net in this article. The cross-entropy loss function is described as follows:

11 $$\begin{aligned} {\text{Loss}}({\text{x}},{\text{c}}) & = - \log \left( {\frac{{\exp ({\text{x}}[{\text{c}}])}}{{\sum\limits_{{\text{j}}} {\exp } ({\text{x}}[{\text{j}}])}}} \right) \\ & = - {\text{x}}[{\text{c}}] + \log \left( {\sum\limits_{{\text{j}}} {\exp } ({\text{x}}[{\text{j}}])} \right) \\ \end{aligned}$$

Among them, $\mathrm {x}=\left[ \mathrm {x}_{0}, \mathrm {x}_{1}, \ldots , \mathrm {x}_{\mathrm {c}-1}\right]$ represents the non-softmax output, and c is the number of class. In this paper, c = 3, $x=\left[ x_{0}, x_{1}, x_{2}\right]$, 0 corresponds to the background, 1 corresponds to OD, and 2 corresponds to OC. Therefore, the loss function in this article is specifically expressed as follows:

12 $$\begin{aligned} {\text {Loss}}(x,3)=-x[3]+\log (\exp (x[0]) +\exp (x[1])+\exp (x[2])). \end{aligned}$$

## Data Availability

The Drishti-GS1 dataset: http://cvit.iiit.ac.in/projects/mip/drishti-gs/mip-dataset2/Home.phphttp://cvit.iiit.ac.in/projects/mip/drishti-gs/mip-dataset2/Home.php

## Abbreviations

OD: Optic disc
OC: Optic cup
CNN: Convolution Neural Network
RSAP: Residual spatial attention path
MSRCR: Multi-scale retinex with colour restoration
ROI: Region if interest
PT: Polarization transformation
